# Residual swin transformer for classifying the types of cotton pests in complex background

**DOI:** 10.3389/fpls.2024.1445418

**Published:** 2024-08-27

**Authors:** Ting Zhang, Jikui Zhu, Fengkui Zhang, Shijie Zhao, Wei Liu, Ruohong He, Hongqiang Dong, Qingqing Hong, Changwei Tan, Ping Li

**Affiliations:** ^1^ College of Mechanical and Electrical Engineering/Modern Agricultural Engineering Key Laboratory at Universities of Education Department of Xinjiang Uygur Autonomous Region/Key Laboratory of Tarim Oasis Agriculture (Tarim University) Ministry of Education, Tarim University, Alar, China; ^2^ Jiangsu Co-Innovation Center for Modern Production Technology of Grain Crops/Joint International Research Laboratory of Agriculture and Agri-Product Safety of the Ministry of Education of China/Jiangsu Province Engineering Research Center of Knowledge Management and Intelligent Service, College of Information Engineer, Yangzhou University, Yangzhou, China; ^3^ Corps in Southern Xinjiang, College of Agronomy, Tarim University, Alar, China; ^4^ Jiangsu Key Laboratory of Crop Genetics and Physiology/Jiangsu Key Laboratory of Crop Cultivation and Physiology, Agricultural College of Yangzhou University, Yangzhou, China

**Keywords:** cotton pests, swin transformer, complex background, deep learning, unmanned aerial vehicles

## Abstract

**Background:**

Cotton pests have a major impact on cotton quality and yield during cotton production and cultivation. With the rapid development of agricultural intelligence, the accurate classification of cotton pests is a key factor in realizing the precise application of medicines by utilize unmanned aerial vehicles (UAVs), large application devices and other equipment.

**Methods:**

In this study, a cotton insect pest classification model based on improved Swin Transformer is proposed. The model introduces the residual module, skip connection, into Swin Transformer to improve the problem that pest features are easily confused in complex backgrounds leading to poor classification accuracy, and to enhance the recognition of cotton pests. In this study, 2705 leaf images of cotton insect pests (including three insect pests, cotton aphids, cotton mirids and cotton leaf mites) were collected in the field, and after image preprocessing and data augmentation operations, model training was performed.

**Results:**

The test results proved that the accuracy of the improved model compared to the original model increased from 94.6% to 97.4%, and the prediction time for a single image was 0.00434s. The improved Swin Transformer model was compared with seven kinds of classification models (VGG11, VGG11-bn, Resnet18, MobilenetV2, VIT, Swin Transformer small, and Swin Transformer base), and the model accuracy was increased respectively by 0.5%, 4.7%, 2.2%, 2.5%, 6.3%, 7.9%, 8.0%.

**Discussion:**

Therefore, this study demonstrates that the improved Swin Transformer model significantly improves the accuracy and efficiency of cotton pest detection compared with other classification models, and can be deployed on edge devices such as utilize unmanned aerial vehicles (UAVs), thus providing an important technological support and theoretical basis for cotton pest control and precision drug application.

## 1 Introduction

Cotton is one of the most important cash crops in the world and occupies an important position in the economic development of China and the world. Cotton may be subjected to various viruses, bacteria, fungi, and insects during its growth, which can have a significant impact on its quality and yield (Chen et al., 2020; Kiruthika et al., 2022). Cotton aphid infections in the seedling stage of cotton can lead to short plants and shriveled leaves; infestation by cotton mirids bugs at the bud stage can lead to bud shedding and sparse boll setting; cotton leaf mite infestation at the boll stage causes leaf, bud, flower, and young boll abscission; causing irreparable damage to growth and yield (Islam et al., 2023; Thivya Lakshmi et al., 2024). Insect-damaged plants usually show visible signs or lesions on leaves, stems, flowers, or fruits, and each type of damaged leaf exhibits unique characteristics (Li et al., 2021; Zhu et al., 2023). Traditional identification methods are still mainly based on manual visual hand checking, the method relies on personal experience, has a strong subjectivity, and low work efficiency (Skelsey et al.,2013; Zhang et al., 2022). Therefore, the accurate identification of cotton insect pests is a key component in the prevention and treatment of cotton pests, as well as reducing the use of pesticides and promoting the development of green agriculture.

In recent years, with the rapid development of agriculture 4.0, the use of advanced information systems and Internet technology can realize a large amount of agricultural data collection, analysis, processing, and realization of providing assistance to the agricultural decision support system (Zhai et al., 2020). In early studies, (Arnal Barbedo, 2013; Manavalan, 2022; Kong et al., 2022) used traditional machine learning and image processing methods to extract pest and disease features for classification and identification. (Haoyu et al., 2010) used the support vector machine(SVM) algorithm with four kinds of kernel functions to process the spectral data and establish the diagnostic model of cucumber pests and diseases to realize the rapid and accurate diagnosis of cucumber pests and diseases, with the highest recognition rate of 98.3%. (Chaudhary et al., 2016). proposed an improved Random Forest Classifier (RFC) method, which combines the forest machine learning algorithm, attribute evaluator method, and instance filtering method to achieve the identification of peanut diseases with a classification accuracy of 97.8%. (Irfan et al., 2019) used Support Vector Machine (SVM) and C4.5 algorithm for disease and pest identification of leaves, stems, fruits of chili peppers and the study showed that the accuracy of the C4.5 based method is higher. However, such an operation requires certain basic knowledge and specialized skills in image processing, which makes it difficult to promote its application in field production.

In recent years, computer vision techniques have been widely used in agriculture, including machine learning (ML), convolutional neural networks (CNN), deep learning (DL) and transfer learning (TL). Deep learning techniques are favored by many scholars in detecting plant pests and diseases (Rezaei et al., 2024; Luo et al., 2023; Reddy et al., 2023). (Ramcharan et al., 2017) utilized cassava disease image dataset and applied transfer learning to train deep convolutional neural networks to achieve the recognition of three diseases and two insect pests in cassava, and the overall accuracy of the best model was tested to be 93%. However, image data with a single background encountered problems in practical applications in agriculture, so (Yanfen Li et al., 2020) proposed a fine-tuned GoogLeNet model for the complex background presented by farmland scenes to realize the recognition of 10 common crop pests. Since the occurrence of pests and diseases is affected by regional, climatic and other factors, there will be uneven data on pests and diseases, (Abbas et al., 2021) and other scholars addressed this problem by using Conditional Generative Inverse Networks (C-GAN) to generate synthetic images of tomato plant leaves, and realized the classification of 10 types of diseases in tomato through DenseNet121 model, with an accuracy rate of 97.11%. Deep learning-based detection methods have greater advantages in terms of detection efficiency, accuracy and application scenarios (Reder et al., 2021; Syed-Ab-Rahman et al., 2022). Deep learning-based models can provide technical support for edge devices, such as unmanned aerial vehicles (UAVs), to enable wide-area pest detection.

The above research has led to a series of successes in identifying plant pests and diseases. However, most of the models based on deep learning are dominated by convolutional neural networks, which are subject to the limitations of the convolutional network itself, such as the field of view of the convolutional neural network is limited by the size of the convolutional kernel, which can lead to the loss of most of the global information in the image (Guo et al., 2022). Recently, (Liang et al., 2021; Liu et al., 2021) applied the Transformer model in the field of natural language processing to the field of computer vision and achieved good results, and proposed a new vision converter, Swin Transformer, to provide a new direction for research in the field of vision. In this study, from the practical application of agriculture, we propose a cotton insect pest classification model suitable for the field environment by combining the image characteristics of cotton insect pests with the problem of small and unbalanced image data of cotton insect pests. In this study, three kinds of cotton insect pests (cotton aphids, cotton mirids, cotton leaf mites) are taken as the research objects, and the leaf images of cotton insect pests under the complex background of the field are collected as the dataset, and Swin Transformer model is chosen as the backbone, and the residual module and skip connection are introduced to construct the classification model of cotton insect pests, and to realize the recognition of cotton insect pests in the field environment.

The main contributions of this paper are summarized as follows:

1. An enhanced version of the standard Swin Transformer architecture, the residual Swin Transformer, is proposed, which is capable of accurately categorizing cotton pest species.
2. In Swin Transformer, 2 residual modules and skip connection are added to improve the recognition accuracy of the model while retaining the excellent design of Swin Transformer. Meanwhile, we verify the effectiveness of the residual Swin Transformer through many experiments.

The subsequent sections are structured as follows: section 2 describes the materials and methods used in this study, section 3 organizes and summarizes the experimental results, section 4 analyzes the results, and section 5 provides conclusions.

## 2 Materials and methods

### 2.1 Materials

#### 2.1.1 Image data acquisition and dataset production

**Figure 1:**
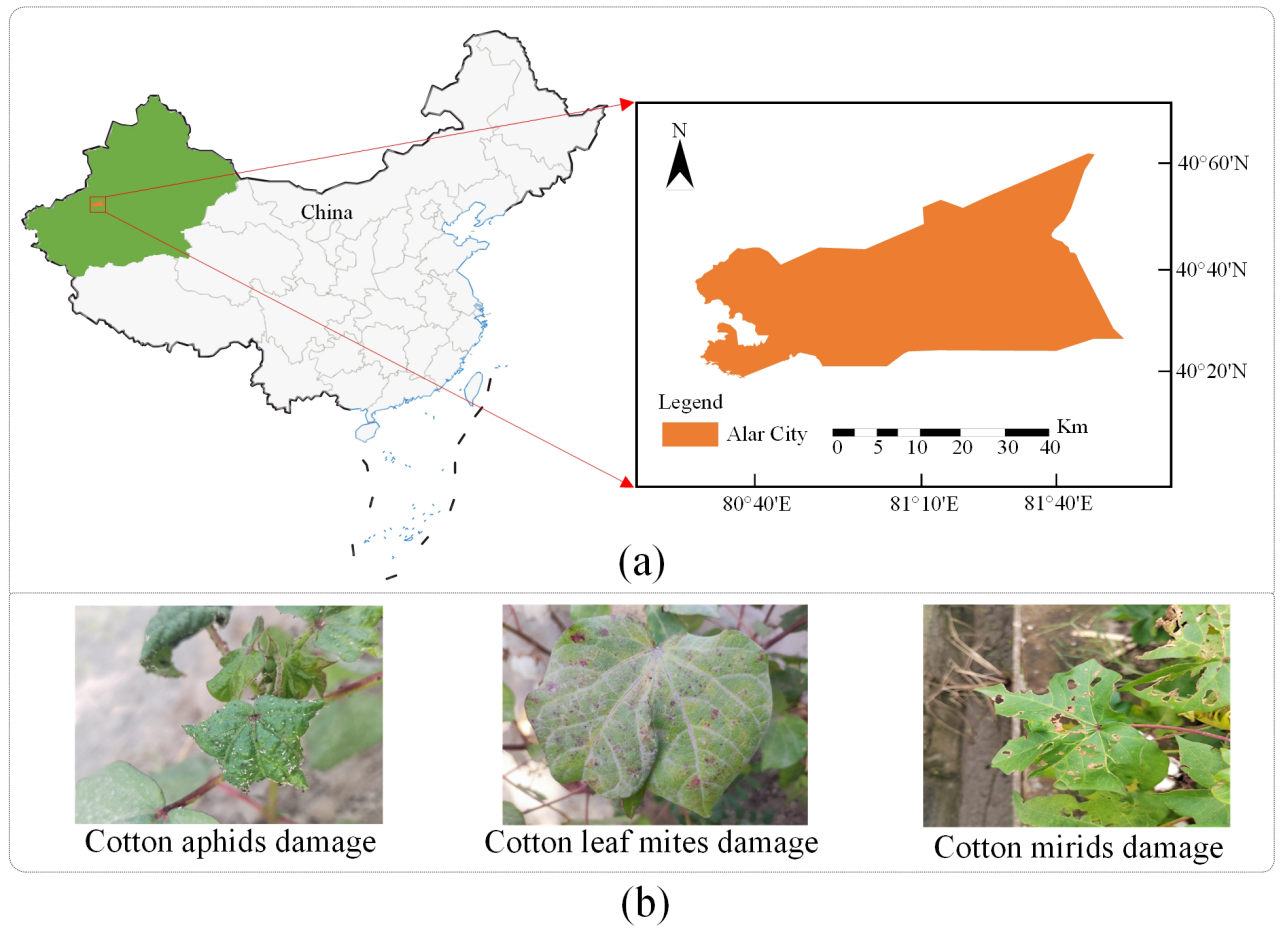
**(A)** Collecting location information; **(B)** Images of cotton pests.

This study collected and constructed a dataset on cotton insect pests for three insect pests, including cotton aphids, cotton leaf mites, and cotton mirids. The dataset was collected in May-August 2023 during the cotton planting period at the cotton experimental field of Tarim University in Xinjiang, China, as shown in **Figure 1A**. The image capturing work was carried out using a mobile device paired with 108MP primary camera + 8MP wide angle camera and the resolution of the acquired image was 2928×2928 pixels. A total of 5581 images were collected under natural environments, 30 cm away from the leaf surface and perpendicular to the leaf surface. Under the guidance of cotton plant protection experts, 2705 usable images were obtained by removing the leaf images of drug-infested wounds, early senescence and other factors, including 1112 images of cotton leaves damaged by cotton aphids, 703 images of cotton leaves damaged by cotton leaf mites, and 890 images of cotton leaves damaged by cotton mirids. Cotton leaves damaged by cotton aphid will appear curled and white or black aphids will appear on the surface of the leaves, on the back and on the stems (Steckel et al., 2021). Cotton leaf mites use stinging mouthparts to suck sap on the back of cotton leaves. When the number of cotton leaf mites on the back is low, yellow and white spots appear on the surface of the leaves; as the number of cotton leaf mites increases, the spots on the surface of the leaves become reddish, and the area of the spots becomes larger and larger (Ferraz et al., 2017). Cotton mirids through the assassination type mouthparts into the cotton plant to suck sap, fresh leaves were damaged at the beginning of a small black spot, with the growth of the leaf stretching after a large number of broken, forming a “broken leaf madness” (Mithal Rind et al., 2021). The samples for each category are shown in **Figure 1B**.

The organized dataset is divided into training set, validation set, and test set in the ratio of 7:2:1 as shown in **Table 1**. The model learns from the images of the training set, extracting features of the data from them, and improves the learning of the model through parameter updating and optimization. By interacting with the validation set, the best model and hyperparameter configurations can be selected to improve the generalization of the model. Finally, the performance of the model is objectively assessed by evaluating its performance on a test set and will be used as an indicator of the final performance of the model.

**Table 1:** Partitioning of cotton image dataset.

Pests	Training Set	Validation Set	Test Set	Total
Cotton aphids damage	779	222	111	1112
Cotton leaf mites damage	493	140	70	703
Cotton mirids damage	624	177	89	890
**Total**	1896	539	270	2705

#### 2.1.2 Image resizing

During this experiment, the original images collected were too large to be used directly for model training. Therefore, the image is resized using Bicubic interpolation algorithm (Keys, 1981). The bicubic interpolation determines the value of the target pixel by calculating the weighted average of the 16 nearest neighbor pixels around the target pixel, which achieves image smoothing and at the same time is able to better preserve the details of the image. The interpolation formula is shown as follows:


$$f\left(i+u,j+v\right)=\sum_{a=-1}^{2}\sum_{b=-1}^{2}f\left(i+a,j+b\right)W\left(a-u\right)W\left(b-v\right)\;\;\;\;$$

where f is the pixel value of the pixel point and u, v are the distance between the pixel points. where W(x) is the interpolating kernel function with the expression as follows:


$$W\left(X\right)=\{\begin{array}{cc} \left(a+2\right){\left|x\right|}^{3}-\left(a+3\right){\left|x\right|}^{3}+1 & \;0<\left|x\right|\leq 1\\ a{\left|x\right|}^{3}-5a{\left|x\right|}^{2}+8a\left|x\right|-4a & \;1<\left|x\right|<2\\ 0 & \;2\leq \left|x\right| \end{array}$$

where a=−0.5 in Equation.

**Figure 2:**
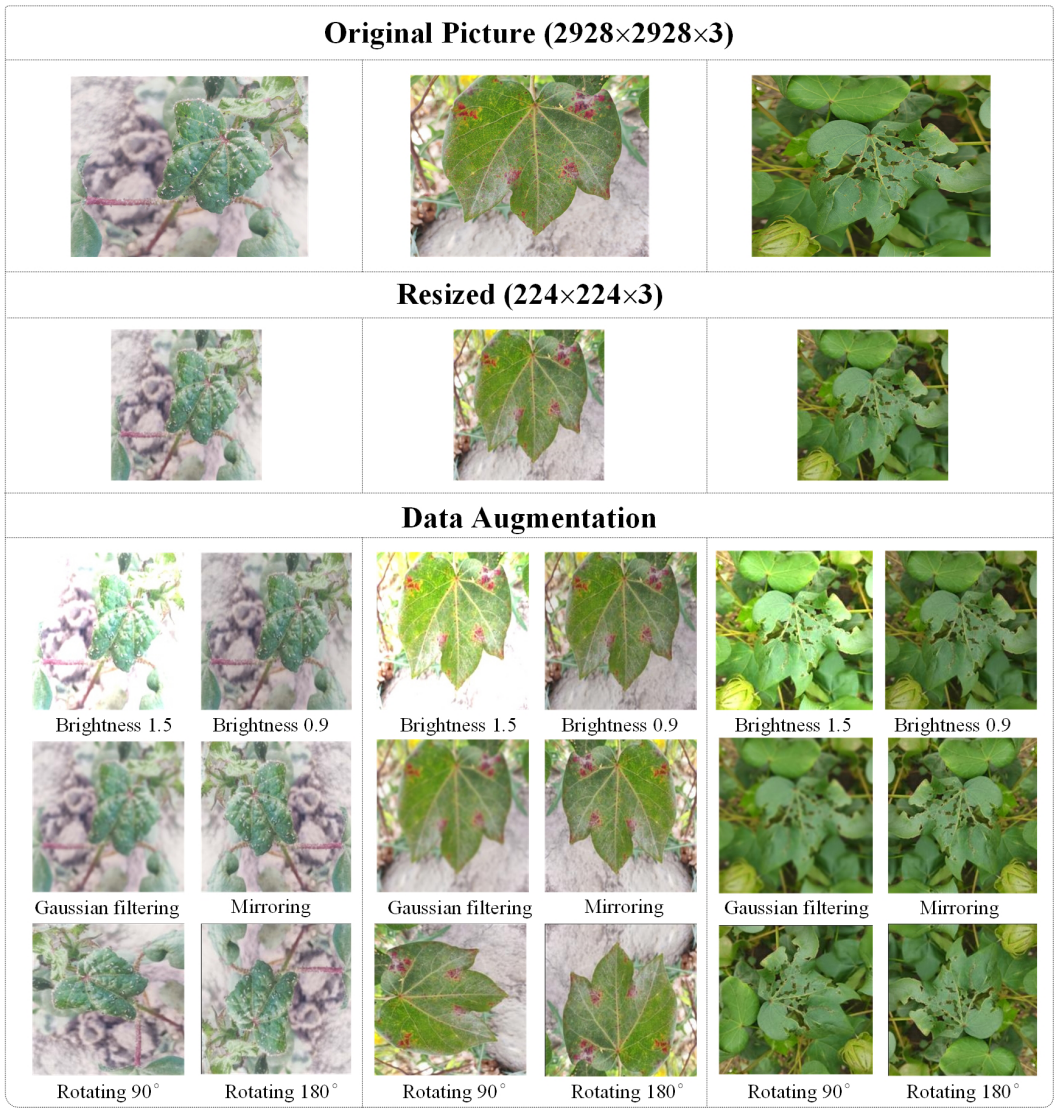
Data preprocessing results.

The size of the processed image by bicubic interpolation is 224×224×3 and the processed image is shown in **Figure 2**.

#### 2.1.3 Image augmentation

In deep learning, training requires large-scale data samples in order to help the model learn a wider range of features and patterns. The collection of cotton pest images is limited by the natural environment and weather conditions, resulting in a limited number of images. In this study, the number of training samples is increased by data augmentation. In order to simulate the growth of leaves in random directions and weather conditions such as sun exposure and cloudy days in a field environment, the data augmentation work was carried out in this study by using the technique of rotating 90°, 180°, mirroring, dimming brightness by 0.9, and brightening brightness by 1.5 images. Considering that the cotton plantation is located in the Tarim Basin, where severe weather with sandstorms often occurs, Gaussian filter image technique is added for image processing so as to simulate the interference of wind and sand. **Figure 2** shows the cotton image after data augmentation. After the data augmentation operation, a total of 16,230 images of cotton pests were obtained, and the partitioning of the augmented dataset is shown in **Table 2**.

**Table 2:** Partitioning of augmented cotton image dataset.

Pests	Training Set	Validation Set	Test Set	Total
Cotton aphids damage	4674	1332	666	6672
Cotton leaf mites damage	2958	840	420	4218
Cotton mirids damage	3744	1062	534	5340
**Total**	11376	3234	1620	16230

### 2.2 Methods

#### 2.2.1 Swin transformer network

Originally proposed by Google in 2017, the Transformer model is mainly used for natural language processing tasks such as machine translation and language modeling (Vaswani et al., 2023). With the successful application of the Transformer model in processing natural language tasks, some scholars have begun to try to apply the model in visual tasks. In 2020, Vision Transformer (VIT) was proposed as a model mainly for image classification tasks (Dosovitskiy et al., 2021). However, VIT suffers from information loss and increased computation, a new Transformer model has been proposed. Swin Transformer improves on VIT by enabling computation in non-overlapping windows through a shifting window scheme, while also allowing cross-window connections for better scalability and efficiency (Liu et al., 2021).

**Figure 3:**
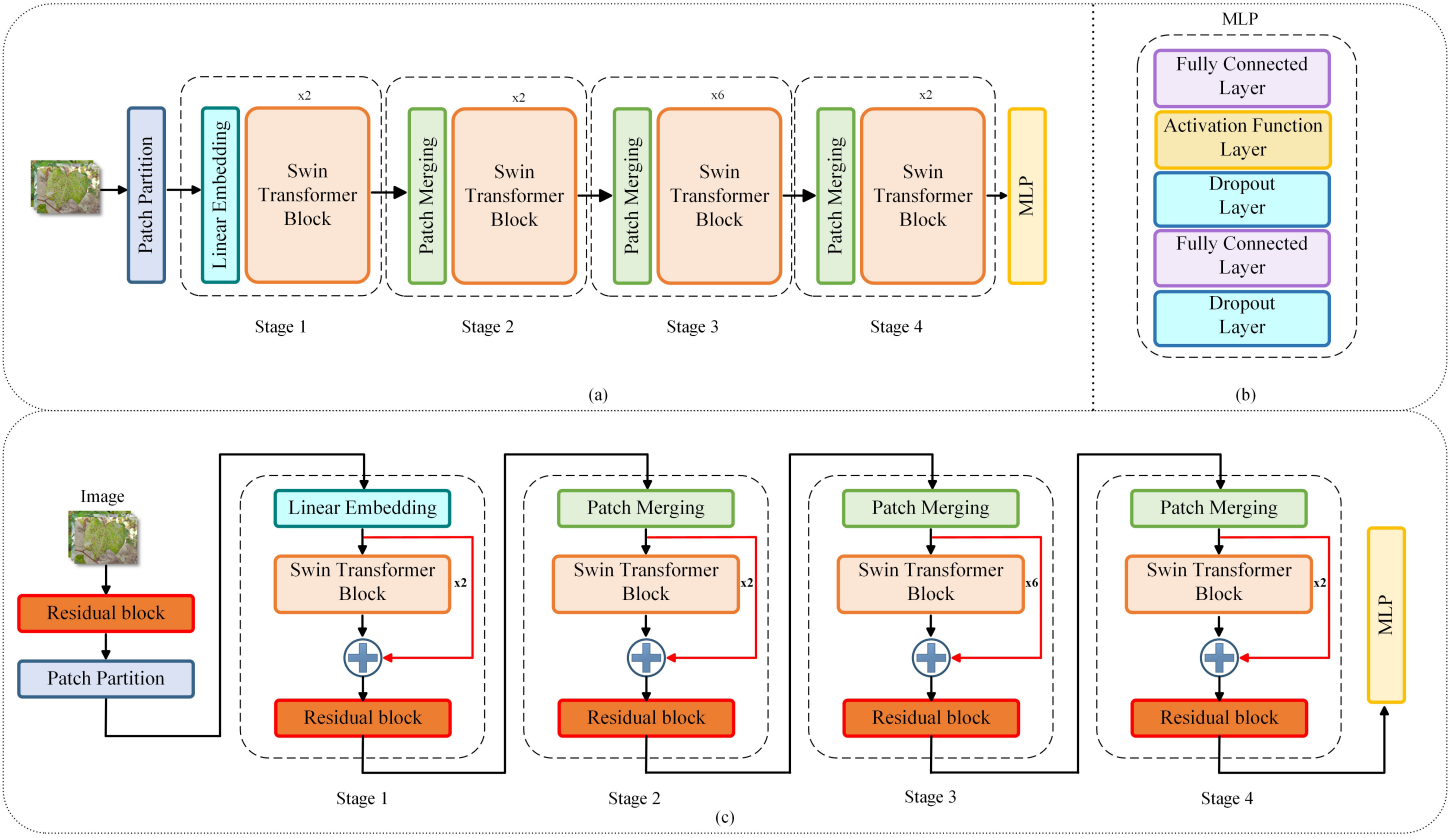
**(A)** Swin Transformer Structure; **(B)** Multilayer Perceptron Structure; **(C)** Improved Swin Transformer structure.

The Swin Transformer model was chosen as the base model for this study. As shown in **Figure 3A**, Swin Transformer consists of the following parts: the Patch Partition, the Swin Transformer Backbone Block, the Classification Head. In this study, the Patch Partition section splits the cotton leaf image into a series of equal-sized image patches. The position of the split cotton image patches is encoded by Linear Embedding, and the position information is embedded into the feature vectors so that the model can perceive the relative positions of the image patches. The Swin Transformer backbone block consists of multiple Transformer encoders containing both Window-MSA and Shifted Window-MSA. Classification Head Part realizes the classification prediction by Multilayer Perceptron network (MLP), the structure is shown in **Figure 3B**. Aiming at the problem that the leaf features of cotton insect pests are small and easy to be confused in the complex background, this study introduces the residual module and skip connection in the Swin Transformer network, and the improved whole model structure is shown in **Figure 3C**.

#### 2.2.2 Residual module

In deep learning, more abstract properties can be extracted as the number of network layers increases. However simply increasing the number of network layers leads to the problem of vanishing and exploding gradients (Zhang et al., 2018; Alaeddine and Jihene, 2023). Because the leaf features of cotton insect pests are relatively small, few in number, and easily confused with the background, the pest features are easily lost in the deep network. In order to solve these problems, this study builds the residual module, which is combined with Swin Transformer network.

**Figure 4:**
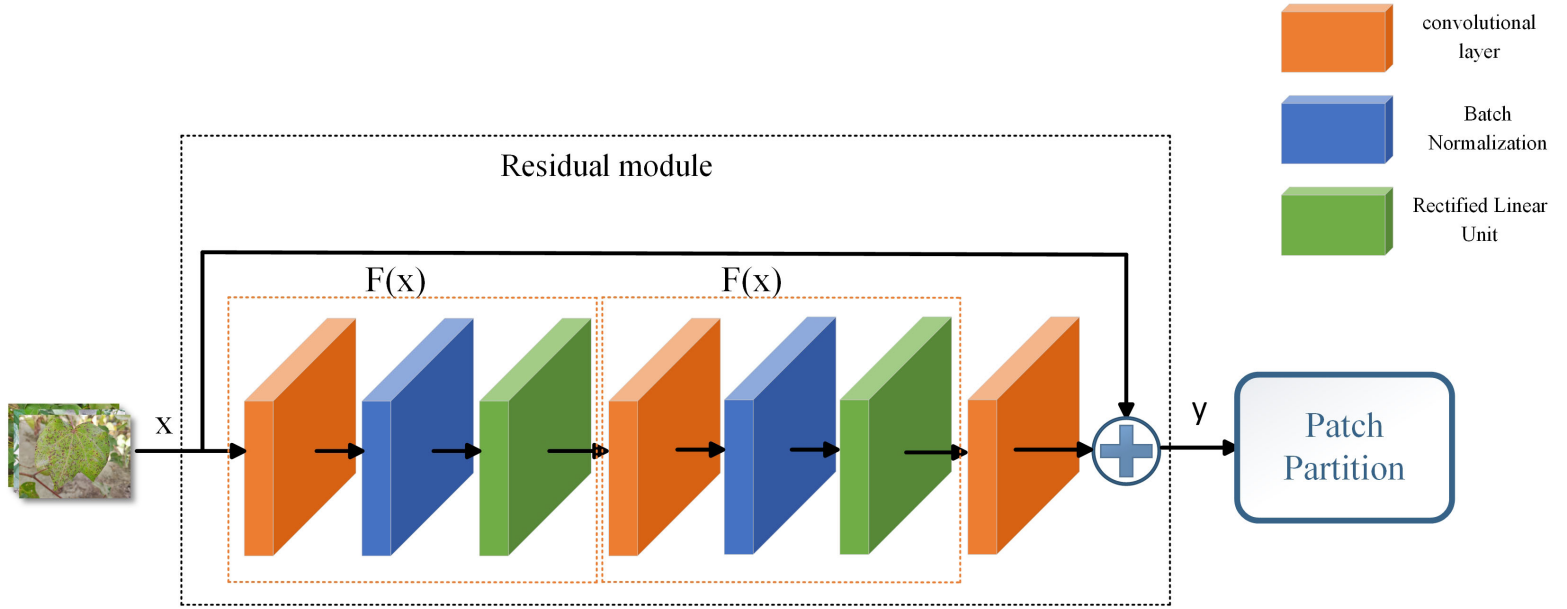
Structure of residual module.


In this study, the residual module is built to enhance the network representation by combining the input features with the output features to preserve the input features, as well as to integrate the classification features of the image. The residual structure is shown in **Figure 4**, including convolutional layer, Batch Normalization, Rectified Linear Unit. Suppose x is the input, σ is the Rectified Linear Unit (ReLU), BN is the Batch Normalization, and y is the output.

F(x)={BN[Conv2d(x)]}

y=x+Conv2d{F[F(X)]}

In this study, the residual module is introduced at two locations respectively, as shown in **Figure 3C**. The first place is located after the image input and before the Patch Partition, where the residual module both preserves the input image features and incorporates classification features to improve the model’s detection of fine leaf features. The second is located between the Swin Transformer blocks and is used in conjunction with the skip connection in 3.3.

#### 2.2.3 Skip connection

Skip connections are commonly used in deep networks to improve information transfer. In deep networks, information is passed from layer to layer, layer by layer. In deep networks, a single sequential delivery method can easily cause gradient vanishing or gradient explosion. Skip connections can be realized to cross over certain layers for information transfer, fusing the output of the previous layer with the input of one of the subsequent layers, increasing the flow of information (Zhang et al., 2023; Ding et al., 2024). Considering the problem that the characteristics of insect pest leaves are small and easy to be lost in the transfer process, this experiment introduces skip connections in stage 1 - stage 4 of the backbone network.

The skip connection can realize the fusion of the underlying features and the higher-level features to retain more high-resolution details and provide more information for the later image classification, thus improving the correct rate of classification. Experimentally, it was found that after adding skip connections, there may be a semantic gap between the two combined feature sets, which is problematic to use directly after fusion. Therefore, in this experiment, the fused data were processed again after fusion. As shown in **Figure 3C**, skip connections are introduced between Swin Transformer blocks, and the data are processed again after fusion by the residual module proposed in 3.2. Experiments have demonstrated that reprocessing the fused data through the residual module after skip connections can improve the whole performance of the network and increase the accuracy of the model. The structure of the original Swin Transformer model is shown in **Figure 3A** and the structure of the improved Swin Transformer model is shown in **Figure 3C**.

#### 2.2.4 Self-attention mechanism

**Figure 5:**
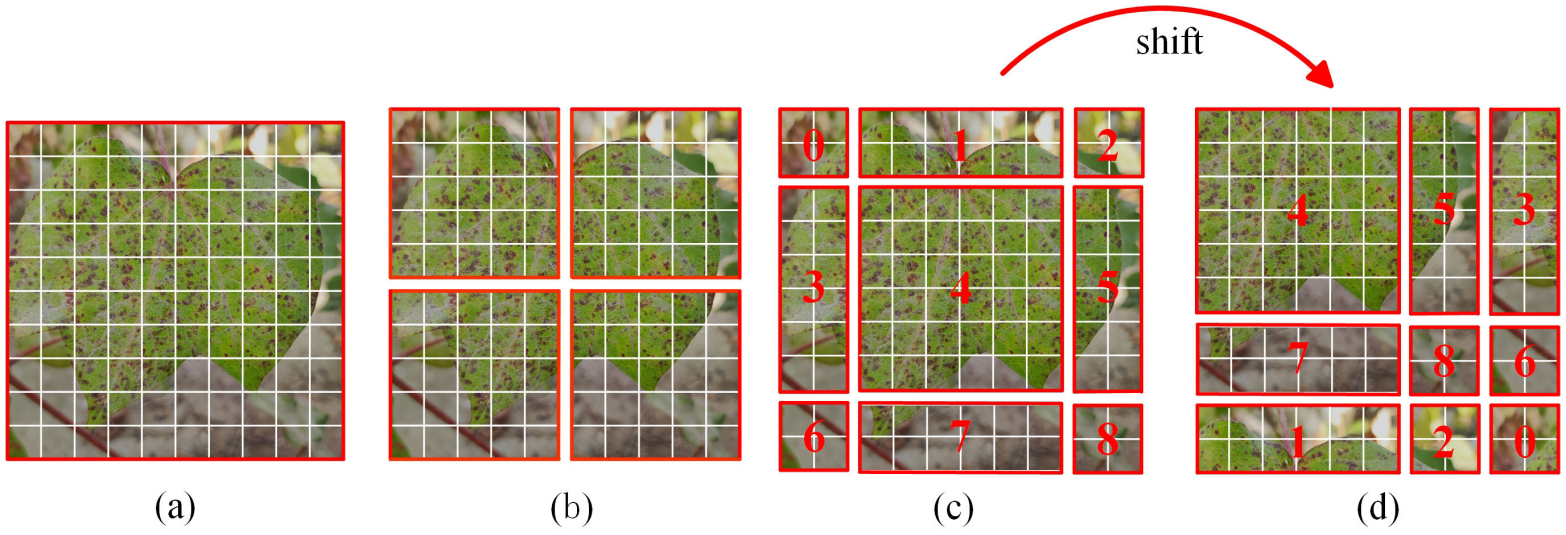
Illustration of Attention Mechanisms.

Self-attention is a key part of the Transformer model in visual tasks. As shown in **Figure 5A**, the input image is segmented into multiple patches, and after recombining them into a set of series, the correlation calculation between each position in the sequence and the other positions is realized through the self-attention. By mapping the input sequence into query ( $W^{Q}$ ), key ( $W^{k}$ ), and value ( $W^{v}$ ) vectors, the similarity, weighted summation, and the correlation of different positions are computed (Vaswani et al., 2023).

The traditional self-attention computes the attention score for the entire image, which suffers from high computational effort. The window self-attention used in this paper was proposed by scholars in 2021, which makes upgrades based on the self-attention and reduces the amount of computation (Liu et al., 2021). As shown in **Figure 5B**, after the image is split into multiple patches, the neighboring patches are integrated into a single window, and the attention score between each window is calculated, thus reducing the amount of computation. The computational complexity of the self-attention (MSA) and the window self-attention (W-MSA) is shown as follows:


$$\Omega MSA=4HWC^{2}+2{\left(HW\right)}^{2}C$$

$$\Omega W-MSA=4HWC^{2}+2M^{2}HWC$$

In order to improve the problem of missing information interactions that can occur between different windows, the Shifted Window self-attention (SW-MSA) is introduced to realize cross-window connection reorganization (Liu et al., 2021). As shown in **Figure 5C**, different from the window self-attention, the shift window self-attention splits the image into 9 pieces of different sizes, which are recombined, and the result of the combination is shown in **Figure 5D**. The combined windows are computed to obtain localized attention.

## 3 Results

### 3.1 Installations and evaluation

Deep learning models require a large number of datasets for training. Therefore, we expanded the cropped 2705 cotton pest images into 16,230 images by data augmentation and randomly divided them into training, validation, and testing datasets in the ratio of 7:2:1. We used adaptive motion estimation (Adam) to automatically optimize the learning rate during deep learning model training with a learning rate of 0.001 and a number of training epochs of 100. To ensure minimal validation loss, we saved the trained model in each epoch. In this study, when the model has finished training, it is validated on the same test dataset using performance metrics such as Precision, Recall, etc. to reveal the detection accuracy of different models. The computer parameters used for model training and validation and the environment configuration for improving the model are shown in **Table 3**. In the classification task, four types appear in the prediction results. True Positive (TP) represents a predicted positive sample that is actually positive and correctly determines the type of disease. False positives (FP) represent samples that were predicted to be positive and were actually negative. True Negative (TN) predicts a negative sample and is actually a negative sample. False negative (FN) represents a negative prediction and a positive actual. The following common classification metrics are commonly used to evaluate the model.

**Table 3:** Computer parameters and improved model environmental resource allocation.

Configuration	Parameter
Operating system	Ubuntu 20.04.6 LTS workstation
CPU	11th Gen Intel® Core™ i7-11700K @ 3.60GHz × 16
GPU	NVIDIA GeForce RTX 3070
Accelerated environment Development environment Language Framework	CUDA 11.4, CUDNN 11.4 PyCharm Python3.7 Pytorch1.8.0 Torchvision0.9.0

Precision rate P : the ratio of the number of samples predicted to be positive and correct to the total number of samples predicted to be positive. The formula is shown as follows:


$$P=\frac{TP}{TP+FP}$$

Recall R : The ratio of the number of samples whose predictions are positive and correct to the total number of samples that are actually positive. The formula is shown as follows:


$$R=\frac{TP}{TP+FN}$$

Specificity S : The ratio of correctly predicted as a negative sample to the actual negative sample. It is an indicator that evaluates the ability to judge negative samples. The specificity was calculated as follows:


$$S=\frac{TN}{TN+FN}$$

$F_{1}$ -measure: weighted summed mean of precision rate P and recall rate R . The accuracy and coverage of the model can be evaluated in a comprehensive manner. Higher $F_{1}$ scores indicate that the model strikes a better balance between precision and recall. The formula is shown as follows:


$$F_{1}=\frac{2\times P\times R}{P+R}$$

Accuracy ( Acc ): The ratio of the number of correctly categorized samples to the total sample number. Accuracy is one of the commonly used evaluation measures. The formula is shown as follows:


$$Acc=\frac{TP+TN}{TP+FP+TN+FN}$$

### 3.2 Experimental results and analysis

#### 3.2.1 Comparison of data augmentation results

Model training and validation were performed on the original and augmented datasets. The experimental results show that using the original model for training and validation on the original and augmented datasets, the accuracy was improved from 0.946 to 0.951. Using the improved model for training and validation respectively, the accuracy was improved from 0.961 to 0.974, which is an improvement of 1.3%. The experimental data proved the effectiveness of the data augmentation operation. Therefore, the rest of the experiments were conducted on the augmented dataset.

#### 3.2.2 Performance comparison of improved modules

The Swin Transformer model is applied to detect cotton insect pests in complex environments in the field, which may be affected by complex environments. Therefore, this paper introduces a residual module and skip connection to improve the detection accuracy of the model. In order to verify the effectiveness of the improvement scheme proposed in this paper, we take the Swin Transformer model as the original model and add different improvement modules respectively, so as to verify the performance of the improved model. One of the residual module1 is mentioned in 3.2 and is located after the picture input. The skip connection and residual module 2 are the ones mentioned in 3.3 of the text and are located between the Swin Transformer blocks and after the skip connection, respectively. The experimental results are shown in **Table 4**, where the accuracy is improved from 0.951 to 0.959 by adding the residual module 1 to the original model. The accuracy is improved by 1.3% and 1.2% by adding skip connection and residual module 2, respectively. Finally, the improved model improves the accuracy from 0.951 to 0.974 compared to the original model, an improvement of 2.3%. Comparison results can prove that the residual module and skip connection introduced in this paper contribute to the model performance.

**Table 4:** Impact of improved modules on model performance.

Swin Transformer	Residual module 1	skip connection	Residual module 2	Accuracy	Parameter/Ten Thousand	Size/MB
√				0.951	2752.09	106.11
√	√			0.959	2752.18	106.12
√	√	√		0.972	2752.18	106.12
√	√		√	0.971	4163.09	159.98
√	√	√	√	0.974	4163.09	159.98

#### 3.2.3 Analysis of training results

**Figure 6:**
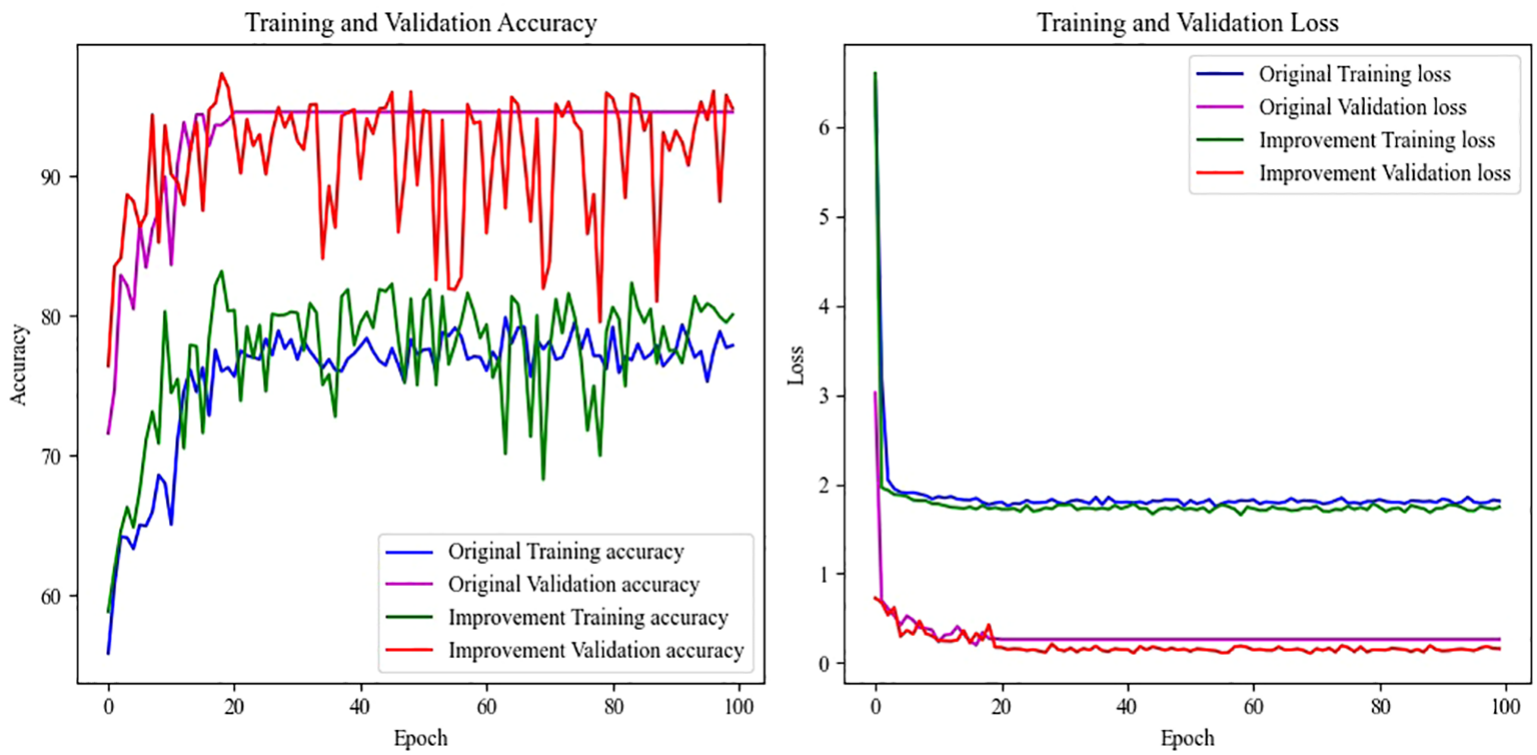
Accuracy- epoch and Loss-epoch curve of model before and after improvement.

The relationship between the training results and the number of epochs before and after the model improvement is shown in **Figure 6**, which shows that the original model has better generalization ability. But after training up to 25 epochs, you can see that the accuracy and loss show a straight line and no longer change. It is possible that the gradient disappears or the gradient explodes. The improved model continues the generalization ability of the original model, solves the problems such as gradient vanishing, and increases the classification accuracy from 0.951 to 0.974, and reduces the loss from 0.2019 to 0.1089.

#### 3.2.4 Analysis of test results

The model weights with the highest rate of accuracy during training are saved and the model is tested on the TEST dataset (1620 sheets). In terms of the accuracy of the model, the accuracy rate reaches 97.4%. Meanwhile, in the process of testing the model, the prediction time for the model was also evaluated, and the average time for single prediction was 0.00434s, and the improved model can judge 230 RGB images in 1s.

The precision, recall, specificity, and $F_{1}\;$ score is calculated and analyzed for each pest by testing. The performance of different pests before and after model improvement is shown in **Table 5**. It can be seen that the improved model has improved and more balanced in most indicators, among which the performance of recognizing cotton aphid damage pictures and cotton mirids damage pictures is not much different, and the accuracy rate is 0.978 and 0.983 respectively. The picture will be slightly inferior in identifying cotton leaf mite damage, but it also reaches 0.957. Combined with the collection of cotton pests, we analyzed that due to the occurrence of cotton leaf mites at the late stage of the cotton planting period, which is in the August-October period in the Xinjiang region, the strong light irradiation and overexposure during the filming, and the lack of characteristics of leaf infestation, led to the difficulties encountered by the model in the detection of infestation characteristics.

**Table 5:** Test results of different insect pests.

Pests	Precision		Recall		Specificity		$F_{1}$	
	Original	Improved	Original	Improved	Original	Improved	Original	Improved
Cotton aphids damage	0.940	0.978	0.934	0.986	0.958	0.984	0.937	0.981
Cotton leaf mites damage	0.882	0.957	0.905	0.943	0.958	0.985	0.893	0.950
Cotton mirids damage	0.987	0.983	0.974	0.983	0.994	0.992	0.980	0.983

#### 3.2.5 Comparison with classical classification networks

Finally, classical classification models were used to train on the same dataset separately. The training results of the classical model and the results of the improved model are shown in **Table 6**. Among them, the classical classification models VGG11, VGG11-BN, Resnet18, and mobilenetv2 achieve high accuracy, which is as high as 0.969, 0.927, 0.952, and 0.949, respectively. The accuracy of VIT and Swin Transformer small, Swin Transformer base in the Transformer series reached 0.911, 0.895 and 0.894 respectively. The model size of the improved model is not the smallest, but it is smaller than the Transformer series and the VGG network, although in the model size increased, the improved model performs the best with an accuracy of 0.974.

**Table 6:** Classification results of different algorithms for cotton insect pests.

Model	Accuracy	Precision	Recall	Specificity	$F_{1}$	Parameter/Ten Thousand	Size/MB
VGG11	0.969	0.977	0.961	0.979	0.967	13286.33	506.84
VGG11-bn	0.927	0.971	0.910	0.959	0.939	13286.88	506.89
Resnet18	0.952	0.957	0.925	0.966	0.936	1168.95	44.66
Mobilenetv2	0.949	0.954	0.962	0.981	0.957	350.48	13.60
VIT	0.911	0.910	0.909	0.922	0.910	8656.76	330.28
Swin Transformer small	0.895	0.895	0.893	0.907	0.894	4960.62	189.79
Swin Transformer base	0.894	0.893	0.892	0.908	0.892	8776.82	335.37
Improved Swin Transformer	0.974	0.973	0.971	0.987	0.972	4163.09	159.98

**Figure 7:**
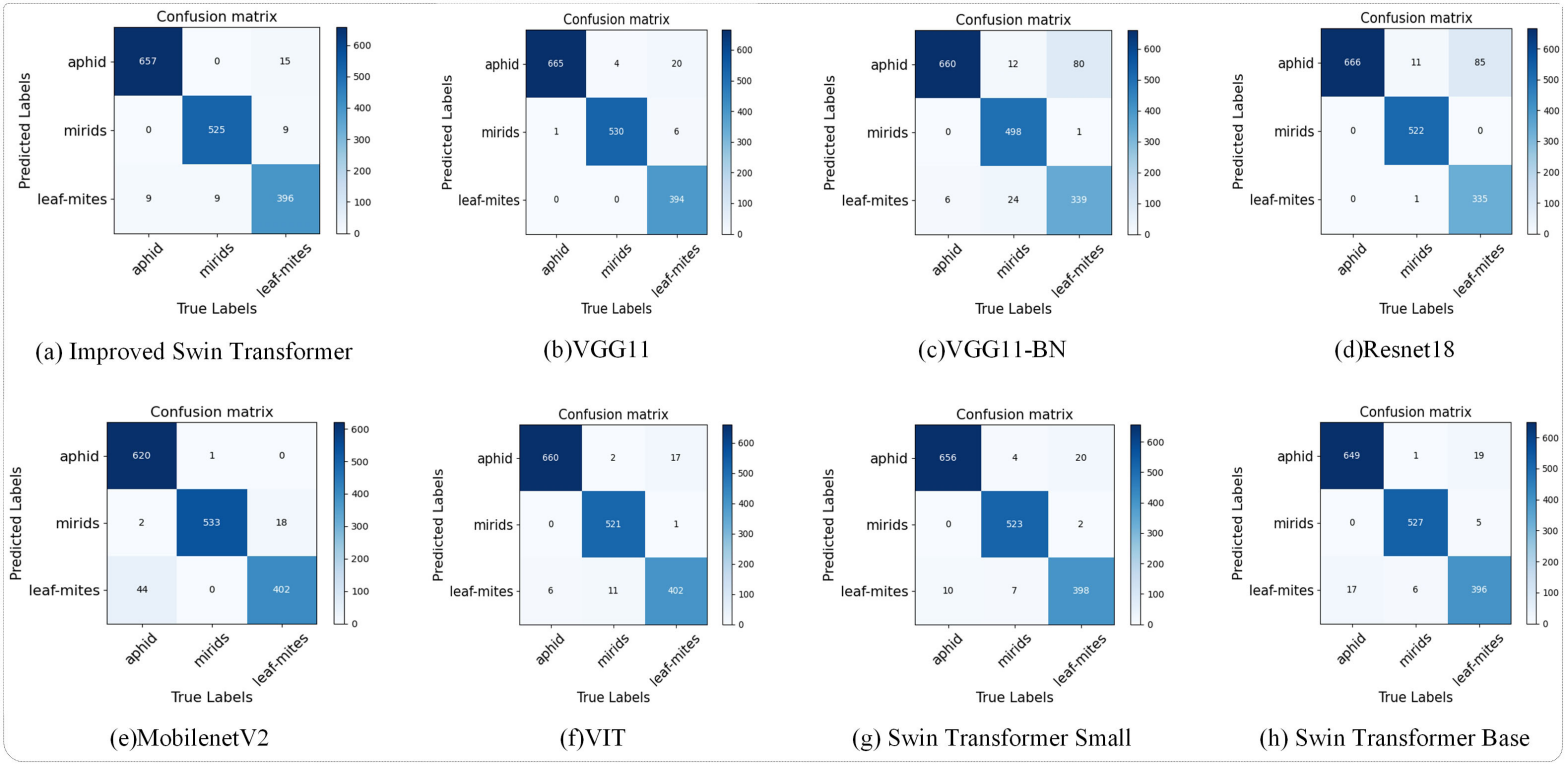
Confusion matrix.

**Figure 8:**
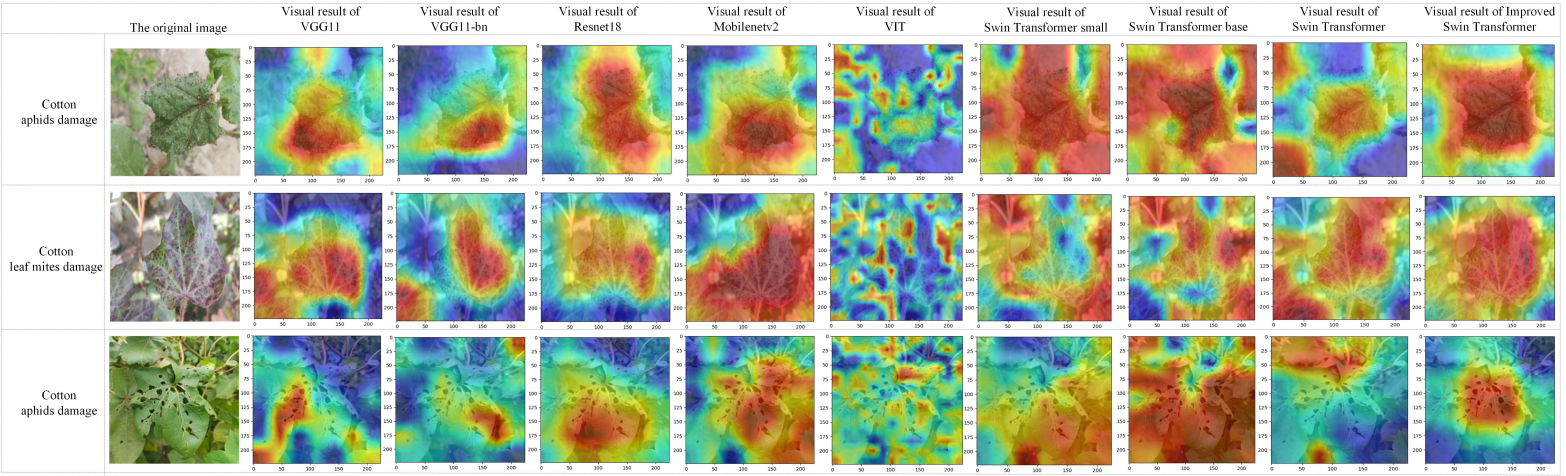
Visual results.

As shown in **Figure 7**, the predictions of the model are visualized with the real labels through the confusion matrix. **Figures 8** shows the prediction results of the improved model and other classical models on the test dataset, which shows that the number of images classified correctly by the improved Swin Transformer model is relatively balanced, indicating that there is no obvious classification preference of the improved model. Among the results of misjudgments, the number of images of cotton infested with cotton leaf mites that were judged to be infested with cotton aphids was higher than the rest of the misjudgments. Comparison of the leaf images of the two infestations revealed that the appearance of brown spots on the leaves of cotton infested with the cotton leaf mite was more similar to the characteristics of the black cotton aphid on the leaves, resulting in a misjudgment.

Compared to images with a single background, images with complex backgrounds increase the difficulty of the model in acquiring leaf features, resulting in some decrease in the accuracy rate. Leaf images with complex backgrounds can contain information such as soil, weeds, and other information, as well as staggered and obscured leaves. The color of the soil in model training is similar to the color of the spots, which can cause learning difficulties; interlocking and partially shaded leaves can cause the model to miss information about the infestation as it learns, leading to less-than optimal learning results.

**Figure 8** shows the recognition visualization results of the improved model and each model in the comparison experiment of 3.2.5. The test results of the three pest images were compared by Grad-CAM visualization (Selvaraju et al., 2016; Chattopadhyay et al., 2018), from left to right, the original image and the visualization results of each model, where Swin transformer is the original model. It can be observed that the improved model can capture more detailed information. For example, as shown in the cotton aphid test results, the improved model focuses on the curly lines at the edge of the leaves. According to the visualization results, the improved model has better detection effect, more accurate localization, and the model has strong anti-interference ability in the complex environment.

## 4 Discussion

In this study, the residual module and skip connection were used to improve the Swin Transformer model, and the improved model could be used for the identification of cotton insect pests in a field environment. Comparing several convolutional neural networks before choosing the benchmark model, it was found that the Swin Transformer model is not limited by the convolutional kernel and can obtain more global information. The Swin Transformer model collects image information through a self-attention mechanism. Effective operation under irregular inputs is achieved by dynamically computing the weight matrix of the self-attention mechanism (Zhu et al., 2022; Zhou et al., 2023). Therefore, Swin Transformer model is chosen as the backbone network in this study.

The inclusion of the residual module helps to enhance the network expressiveness and improve the model classification accuracy. Compared with single background images, cotton pest images in complex backgrounds contain information such as soil, weeds, and so on. There are also problems such as plants occluding each other, which increases the difficulty of obtaining classification features. Feature extraction and fusion of the input information through the residual module preserves the input information and enhances the classification features, thus improving the classification accuracy (He et al., 2024; Wu et al., 2024). Due to the cotton pest early leaf characteristics of small, mostly yellow, red and other small spots on the edge of the leaf, and cotton planting areas are windy and sandy, it is easy to cause the characteristics of the disease spots and complex background confusion. It is easy to lose the insect pest features during the model training process, thus affecting the classification accuracy of the model. In deep networks, skip connections can realize the transfer of information across layers, increase the mobility of information, and solve the problem that pest features are easy to be lost during the training process (Li et al., 2023; V and Kiran, 2022). The results proved that the accuracy of the improved model increased from 94.62% to 97.38%, and the improved model had no obvious classification preference for the three cotton insect pests with balanced classification results.

In recent years, many researchers have used deep learning techniques to study cotton pests and diseases with extensive, high-quality results. As shown in **Table 7**, many researchers used convolutional neural network as the backbone network, optimized and improved it to make it more suitable for cotton pest and disease detection, and finally realized the identification of cotton pests and diseases. Among them, the model proposed by (Islam et al., 2023) had the highest accuracy of 98.70%, but the model could only determine whether cotton was diseased or not, and could not accurately determine the type of pest or disease. The model proposed in this study had the highest accuracy of 97.38% compared to the remaining three models that can determine the type of diseases and pests.

**Table 7:** Performance comparison of the proposed model with previous studies.

Reference	Types of Pests and Diseases	Model	Accuracy
Islam et al., 2023	Healthy leaves +Diseased leaves	Xception	98.70%
Zhang et al., 2022	3 diseases + 2 pests	Improved YOLOX	94.60%
Li et al., 2024	4 diseases + 1 pests + healthy leaves	Improved YOLOv8s	89.90%
M. et al., 2021	2 diseases + 1 pests + healthy leaves	CNN	96.40%
Proposed method	3 pests	Improved Swin Transformer	97.40%

**Figure 9:**
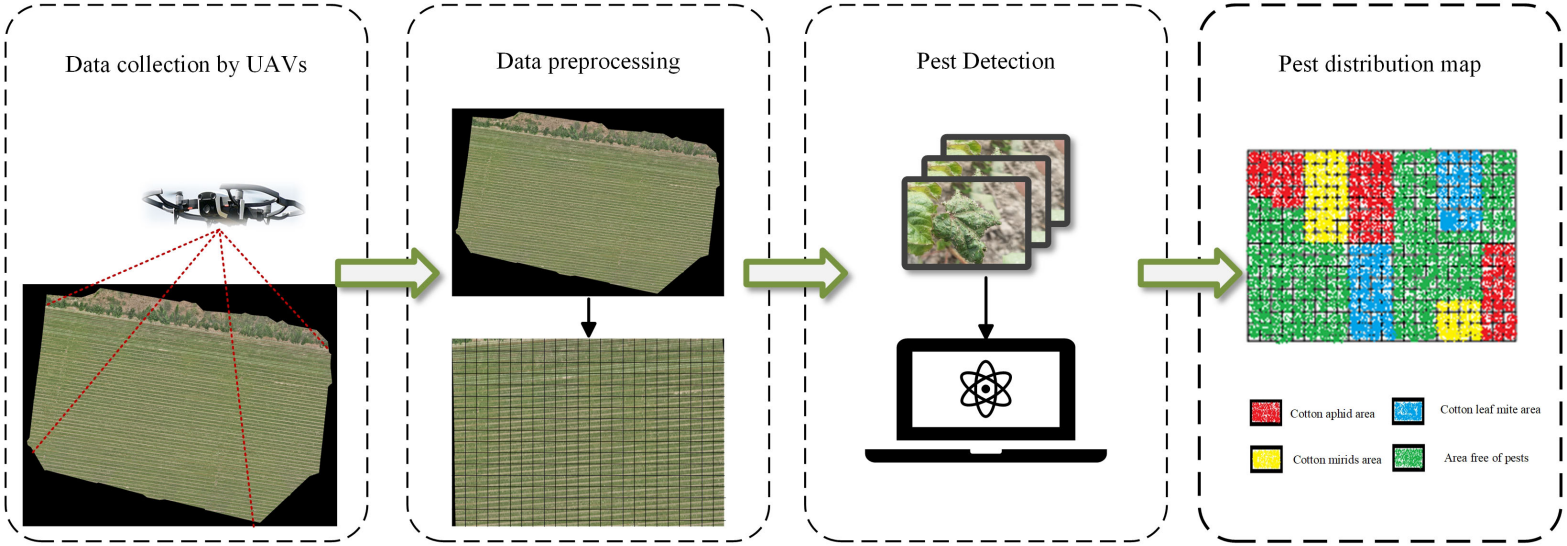
Pest Distribution Mapping Process.

Deep learning techniques had some success in detecting plant leaf diseases (Liang et al., 2019; Bhujel et al., 2022; Nandhini and Ashokkumar, 2021), with some models achieving classification results almost close to 1 on the Plant Village dataset. However, the photos collected in practical applications contain complex backgrounds such as soil and weeds, and there is also the problem of occlusion, which can lead to a decrease in classification accuracy. Therefore, it is more practical to use complex background image data for model training (Wang et al., 2024; Davidson et al., 2024). With the rapid development of UAVs technology and spectral technology, UAVs have attracted the attention of many scholars by showing convenience and safety in the application of large-scale detection of plant pests (Zhang and Zhang, 2023; Sato et al; Zhang et al., 2024). (Xiao et al., 2021) achieved accurate and timely detection of Fusarium powdery mildew in wheat by combining hyperspectral images captured by utilize unmanned aerial vehicles (UAVs) with ground data and selecting the optimal window size of the gray-level co-occurrence matrix (GLCM) to extract texture features. However, the high cost of hyperspectral equipment makes it difficult to popularize its use on a large scale. (Ma et al., 2024) realized the detection of cotton verticillium wilt disease by combining multispectral image data with ground sampling data using simple linear regression (LR) and multiple linear regression (MLR) methods. However, compared to the above models, deep learning-based models are more advantageous in terms of inter-temporal migration. The improved Swin Transformer model does not require manual image processing to achieve accurate identification of cotton insect pests in the field environment, which reduces the influence of human factors to a certain extent and improves the applicability of the model. The edge device could perform pest detection on the captured images by calling the cotton pest model in the cloud server, and will realizes the cotton pest distribution map finally. The whole process is shown in **Figure 9**, where the cotton image acquisition work is carried out by means of a UAV, the acquired utilize unmanned aerial vehicles (UAVs) images are cropped into multiple image segments, which are uploaded to a cloud server for cotton pest detection, and the detection results are annotated using equal-sized patches with different colors, and the detection results are spliced together in accordance with the cropping order to ultimately generate a pest distribution map. Based on the pest distribution map, end users can generate application prescription maps according to the medication guide, which provides technical support for precise and variable medication application and promotes the intelligent development of agricultural diseases.

## 5 Conclusions

In order to realize the accurate identification of cotton insect pests in complex environments, a cotton insect pest detection model based on improved Swin Transformer was proposed in this study, and the main conclusions are as follows.

Aiming at the characteristics of small and unbalanced samples of cotton pest data, the data set was expanded by digital image processing, and the residual module and skip connection were introduced, which effectively improved the model accuracy from 94.62% to 97.38%. The results of the study proved that the improved model can realize the recognition of cotton insect pests in the field environment.

Through the test of the proposed model, the average single prediction time is 0.00434s, which can complete the judgment of 230 pictures of cotton pests within 1s. It can provide technical reference for the utilization of edge devices such as utilize unmanned aerial vehicles (UAVs).

Based on the results of the current research, the proposed model has great potential for cotton pest detection. However there are several issues that need to be considered in the follow-up study. (1) This study only focuses on the detection of cotton insect pests and ignores the detection of cotton diseases, so the detection of cotton diseases needs to be included in the subsequent study. (2) The size of the improved model is 159.98 MB, which needs to be combined with the computational power of the edge devices in the subsequent research, focusing on the lightweight model, to achieve the application of the equipment in the field, such as the precise application of medicine, to solve the actual production problems in the field.

## Data Availability

The raw data supporting the conclusions of this article will be made available by the authors, without undue reservation.
